# Biomarkers associated with cardiovascular disease in patients with early rheumatoid arthritis

**DOI:** 10.1371/journal.pone.0220531

**Published:** 2019-08-05

**Authors:** Anna Södergren, Kjell Karp, Christine Bengtsson, Bozena Möller, Solbritt Rantapää-Dahlqvist, Solveig Wållberg-Jonsson

**Affiliations:** 1 Department of Public Health and Clinical Medicine/Rheumatology, University of Umeå, Umeå, Sweden; 2 Wallenberg Centre for Molecular Medicine (WCMM), Umeå University, Umeå, Sweden; 3 Department of Surgical and Perioperative Sciences, University of Umeå, Umeå, Sweden; 4 Department of Rheumatology, Kristianstad Hospital, Kristianstad, Sweden; 5 Department of Rheumatology, Sunderby Hospital, Luleå, Sweden; Medizinische Universitat Graz, AUSTRIA

## Abstract

**Objectives:**

Patients with rheumatoid arthritis (RA) have an increased mortality and morbidity due to cardiovascular disease (CVD). In this prospective 5-year follow up of patients with RA, we analysed several biomarkers, known to be associated with atherosclerosis and/or inflammation in the general population. The aim of this study was to find out whether the RA-disease *per se* affect these biomarkers and if those could be associated with the progression of atherosclerosis, as measured by intima media thickness (IMT) among patients with early RA.

**Methods:**

Patients from northern Sweden diagnosed with early RA, are consecutively recruited into an ongoing prospective study on CVD comorbidity. A subgroup of patients, aged ≤60 years (n = 71) was included for ultrasound measurements of IMT at inclusion (T0) and after 5 years (T5) together with age-sex-matched controls (n = 40). The patients were clinically assessed. Blood was analysed for lipids, ESR and CRP and several biomarkers known to be associated with atherosclerosis in the general population.

**Results:**

At T0, the patients with RA had significantly lower levels of MIF and significantly higher levels of interleukin (IL)-18 and MIC-1 compared with controls. At T5, the patients with RA had significantly higher levels of pentraxin3, MIC-1, TNF-R2, ICAM-1, VCAM-1 and endostatin compared with controls. At T0 the levels of MPO correlated with DAS28, sCD40L with CRP and IL-18 with systolic blood pressure and Reynolds risk score. Using PLSR on a CVD-panel analysed with multiplex immunoassay, the patients with RA could be correctly classified into those who had a worsening in their IMT over the five years or not. Here, MMP3 was identified as influential.

**Conclusions:**

This study indicates that the RA disease itself could affect several of the biomarkers in this study, and possibly also the processes involved in the development of atherosclerosis.

## Introduction

Patients with rheumatoid arthritis (RA) have an increased atherosclerosis compared with the general population [1–5]. Atherosclerosis is now recognised as an inflammatory disease *per se*, and the two diseases, atherosclerosis and RA, are considered to share many similarities [6], albeit the link between them is, as yet, not evident. Even though, all stages of the atherogenic process appear to be enhanced in RA, all the way to increased incidence of cardiovascular disease (CVD) events [7, 8].

Sub-clinical atherosclerosis precedes CVD and an increased intima media thickness (IMT), measured by ultrasonography, is regarded to be an early indicator of a generalized atherosclerosis [9, 10]. We, and others, have previously shown that patients with established RA have a premature atherosclerosis as measured by an increased IMT of the common carotid artery (CCA) compared with controls [11–13]. However, this ultrasound measured indicator is not easily available and, further, has disadvantages like lack of possibility to detect the very early phases of the development of atherosclerosis. Therefore, to increase both the benefits of health and cost effectiveness, it is important to enhance the accuracy of the identification of the individuals at risk for increased atherosclerosis and subsequently CVD. In recent years, an interest has been growing to identify new molecular mechanisms associated with the developmental process of atherosclerosis [14]. The identification of such new biomarkers may improve the prediction of cardiovascular events and death.

In an inception cohort of patients with RA, we analysed several biomarkers, known to be associated with atherosclerosis and/or inflammation in the general population, and also known to be associated with subclinical atherosclerosis and traditional CVD risk factors. The aim of this study was to find out whether the RA-disease *per se* affects biomarkers known to be associated with atherosclerosis in the general population. Further, to find out whether these biomarkers are associated with the progression of subclinical atherosclerosis among patients with early RA.

## Material and methods

### Patients and controls

As described in detail previously [15–17] this study is a part of a continuing structured programme involving patients with early RA from Northern Sweden for the prospective analysis of the development of CVD using the nationwide Swedish Rheumatology Quality Register (SRQ). All eligible patients with newly diagnosed RA (*i*.*e*., fulfilling the ACR criteria) [18] and being symptomatic for no longer than 12 months are continuously enrolled into the register. Between 2000 and 2004 all patients under 60 years of age from the three most northern counties of Sweden were consecutively invited to participate in an extended survey on CV morbidity. Five years after inclusion into the study (T5), 71 of the 79 patients with RA originally included were willing to participate in the follow-up study, and 40 of the original 44 controls were reassessed. The controls (one control for two patients, but in 13 cases one control per patient) were matched for age (±5 years) and sex. Descriptive data in patients with RA and controls are presented in Table 1 and have been published previously in more detail [15]. The DMARDSs the patients received at T0 were; methotrexate in 37 cases, sulphasalazine in 10, oral gold in one, eight patients were receiving a combination of methotrexate and hydroxychloroquine phosphate, three a combination of methotrexate and sulphasalazine, two a combination of methotrexate, sulphasalazine and hydroxychloroquine phosphate and one had a combination of methotrexate, sulphasalazine and etanercept. During the five years of follow up 66 patients had been treated with methotrexate (mean number of months = 47), 36 with sulphasalazine (mean number of months = 31), 20 with hydroxychloroquine phosphate (mean number of months = 20), 11 with gold salt (mean number of months = 37), 13 with anti-TNF (mean number of months = 14), one with rituximab for 24 months and 14 with other DMARDs (mean number of months = 21). Some of the patients were treated with a combination of these drugs. All individuals gave their oral and written consent in accordance with the Declaration of Helsinki. The study was approved by the Regional Ethics Committee of Umeå University, Umeå, Sweden.

**Table 1:** Demographic data, traditional cardiovascular risk factors and measures of disease activity in 71 patients with early RA and in age and sex-matched controls evaluated at both baseline (T0) and after 5 years (T5). Data are expressed as mean value (standard deviation) if not stated otherwise.

	RA (n = 71)		Controls (n = 40)	
	At T0	At T5	At T0	At T5
Female, n (%)	61 (86)		32 (80)	
Age, years	51.5 (10.7)		48.1 (10.9)	
Duration of symptoms, months	16.1 (6.7)		—	
Rheumatoid factor, n (%)	48 (67)		—	
Intima media thickness, mm	0.52 (0.13)	0.58 (0.13)***	0.54 (0.13)	0.60 (0.12)***
Systolic blood pressure, mmHg	123.5 (14.4)	126.3 (13.9)*	117.7 (11.3)	124.1 (12.1)***
Cholesterol, mmol/L	5.5 (0.9)	5.3 (1.0)	5.3 (1.1)	5.6 (1.1)
BMI, kg/m^2^	25.8 (4.0)	25.7 (4.5)	25.1 (4.9)	25.2 (4.2)
ESR, mm/h	17.5 (16.2)	15.6 (11.9)	—	—
CRP, mg/L	11.9 (10.8)	7.7 (7.2)**	—	—
DAS28	3.5 (1.4)	3.1 (1.5)†	—	—
SCORE ^[^^19^^]^	0.9 (1.3)	1.4 (1.7)***	0.6 (0.7)	1.6 (1.9)***
Reynolds Risk Score ^[^^20^^]^	2.2 (3.3)	2.7 (3.3)	—	—

* p-value <0.05,

** p-value<0.01,

***p-value<0.001 compared to T0

### Physical examination and surveys

All patients were examined clinically at their inclusion into the study and regularly thereafter for disease activity according to DAS28 [21]. The examinations have been previously described in detail [15]. Moreover, European Systematic Coronary Risk Evaluation (SCORE) [19] and Reynolds Risk Score [20] were calculated at both T0 and T5. These compound measures of CVD risk factors indicate the risk of death due to CVD (SCORE) and the risk of a CV event (Reynolds Risk Score) during the next 10 years. In addition to the traditional CVD risk factors, the Reynolds Risk Score also includes a measure of inflammation, *i*.*e*. C-reactive protein (CRP). When calculating the Reynolds Risk Score, all patients were regarded as being non-diabetic due to a lack of information regarding haemoglobin A1c concentrations.

### Blood sampling

As described in detail previously [15–17] all patients and controls donated a blood sample at the time of both occasions for the ultrasound measurements, *i*.*e*., at T0 and T5, and serum was stored at -80°C. After thawing, serum concentrations were measured using commercial sandwich enzyme- linked immunosorbent assays (ELISAs) of pentraxin 3 (ptx3) (R&D Systems, Abingdon, UK), interleukin (IL)-18 (MBL, Woburn, MA, USA) and macrophage inhibitory cytokine 1 (MIC-1) (BioVendor, Brno, Czech Republic). Serum concentrations of IL-6, monocyte migration inhibitory factor (MIF), myelopreoxidas (MPO) and soluble CD40 ligand (sCD40L) were measured using multiplexed Procarta immunoassays based on the Luminex technology (Affymetrix Inc). Serum concentrations of tumour necrosis factor receptor 1 and 2 (TNF-R1 and TNF-R2), endoglin, intercellular adhesion molecule 1 (ICAM-1), vascular adhesion molecule 1 (VCAM-1) and endostatin were analysed using commercial ELISAs (R&D Systems, Minneapolis, MN, USA). As a pilot study 19 patients with RA and 10 matched controls, a multiplex immunoassay was used for analysing 92 biomarkers potentially associated with cardiovascular disease (Proseek Multiplex CVD I, Olink Bioscience AB, Uppsala, Sweden). Rheumatoid factor (RF), soluble C-reactive protein (CRP; mg/L) and ESR (mm/h) were measured at baseline according to routine methods. Blood was also drawn after an overnight fast for measurement of blood lipids, *i*.*e*., cholesterol (mmol/L), high-density lipoproteins (HDL; mmol/L) and triglycerides (mmol/L), using routine methods at each of the participating hospitals.

### Ultrasound investigations

The patients with RA were included as soon as possible following a diagnosis of RA (T0), and were examined by ultrasound at mean (SD) of 16.2 (±6.6) months after the primary symptom of RA. The ultrasound examinations at the follow up (T5) were performed at mean (and median) 60 (SD 1.2; range 55–64) months after the initial examination. All examinations were performed by the same experienced investigator and described in detail in previous publications [15, 17].

### Statistics

Differences in variables between patients with RA and matched controls were analysed using simple conditional logistic regression analyses. Comparisons within the RA patient group or within the control group were performed using the Mann-Whitney U-test, or for comparisons over time, the Wilcoxon paired test. Simple and multiple linear regression analyses were used to identify certain variables associated with the different biomarkers. Correlations were tested with Spearman rank correlation or Pearsons correlation, whenever appropriate. For the results from the multiplex immunoassay, first a partial least squares regression (PLSR) was performed, with two components, where the X-variables are scaled by dividing each variable by its standard deviation. Using the scores from the PLSR a k-means clustering with two centers (2-means) (with the Hartigan-Wong algorithm) was performed in order to discriminate between the two groups. The result was evaluated by plotting the scores from the PLSR, that is using the scores as y and x coordinates. Each observation was assigned a colour (red or blue) according to the cluster assigned to that observation. The numbers represent the true group of the observation, that is ideally all observations with one number should have the same colour. Based on results from previous publications, calculations showed that a sample size of 26 in each group would render 95% power to detect a difference in IMT of 0.1mm and SD of 0.1 mm [15]. For statistical analyses, p-values <0.05 were considered statistically significant. Occasional missing values were regarded being at random. All calculations, except the PLSR, were made using SPSS 21.0 (SPSS Inc, Chicago, US).

## Results

### Patients with RA compared with controls

**Fig 1:**
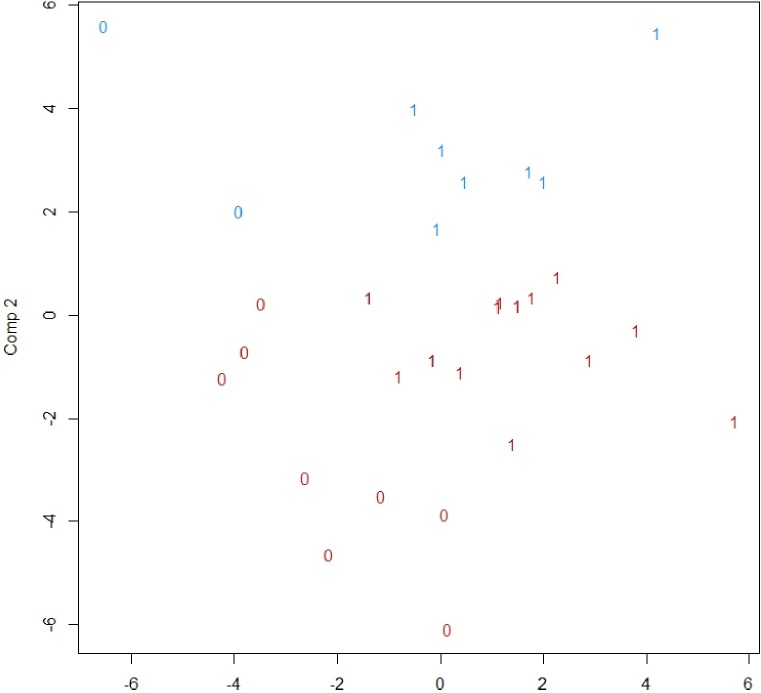
Classification by k-means as well as the true class for an observation for patient with RA or control, in 19 patients with RA and 10 controls. The numbers indicate the true class and the colour the class according to the classification.

At T0, the patients with RA had significantly lower levels of MIF and significantly higher levels of IL-18 and MIC-1 compared with controls (Table 2). At T5, the patients with RA had significantly higher levels of pentraxin3, MIC-1, TNF-R2, ICAM-1, VCAM-1 and endostatin compared with controls (Table 2). The biomarkers included in the multiplex CVD-panel did not separate patients with RA from controls (Fig 1).

**Table 2:** Concentrations of biomarkers important for CVD in 71 in patients with early RA and in 40 age and sex-matched controls evaluated at both baseline (T0) and after 5 years (T5). Data are expressed as mean value (standard deviation).

	RA		Controls	
	At T0	At T5	At T0	At T5
IL-6 (pg/mL)	13.2 (22.0) n = 70	14.4 (27.6) n = 66	6.1 (3.3) n = 38	6.9 (2.5) n = 40
MIF (pg/mL)	504.5 (328.6) xx n = 70	398.7 (152.3) * n = 66	882.4 (469.9) n = 38	496.5 (164.9) *** n = 40
MPO (pg/mL)	212.0 (116.6) n = 70	173.8 (77.3) * n = 66	207.4 (109.1) n = 38	178.5 (56.8) n = 40
sCD40L (pg/mL)	21.9 (7.3) n = 70	22.6 (7.0) n = 66	23.6 (6.5) n = 38	40.4 (56.4) n = 40
pentraxin3 (ng/mL)	1.16 (1.0) n = 66	1.00 (0.5) xx n = 63	1.3 (0.4) n = 35	0.69 (0.3)*** n = 37
IL-18 (pg/mL)	344.4 (111.2) x n = 70	356.2 (118.4) n = 66	286.2 (94.4) n = 38	303.4 (92.1) n = 39
MIC-1 (ng/mL)	1.8 (1.2) x n = 70	1.9 (0.9) xx n = 66	1.2 (0.4) n = 38	1.3 (0.4) n = 40
TNF-R1 (ng/mL)		0.9 (0.7) n = 67		0.8 (0.6) n = 40
TNF-R2 (ng/mL)		7.4 (6.9) xxx n = 67		4.4 (1.1) n = 40
Endoglin (ng/mL)		93.6 (12.5) n = 67		11.6 (13.2) n = 40
sICAM-1 (ng/mL)		189.5 (40.5) x n = 67		178.5 (30.6) n = 40
sVCAM-1 (ng/mL)		365.7 (94.2) x n = 67		325.7 (8.3) n = 40
Endostatin (ng/mL)		121.9 (21.1) x n = 67		111.9 (21.2) n = 40

* p<0.05 compared to T0

**p<0.01 compared to T0

***p<0.001 compared to T0

x p<0.05 compared to controls

xx p<0.01 compared to controls

xxx p<0.001 compared to controls

### T5 compared with T0

The patients with RA had significantly lower levels of MIF and MPO at T5 compared with at T0 (Table 2). Among the controls, a statistically significant change between T0 and T5 was evident for MIF and pentraxin-3 (Table 2).

### Correlations at T0

Among the patients with RA there were some correlations between the different biomarkers at T0 and markers of disease activity in RA or traditional CVD risk factors; MPO with DAS28 (r = -0.4, p = 0.001), sCD40L with CRP (r = 0.3, p<0.01) and IL-18 with systolic blood pressure (r = 0.3, p<0.05) as well as with Reynolds risk score (r = 0.4, p<0.05).

### Correlations between the different biomarkers

The correlations between the different biomarkers are presented in S1 Table.

### Correlations at T5

Also after 5 years of follow up there were correlations between the different biomarkers and markers of disease activity or CVD risk factors; MPO with BMI (r = 0.3, p<0.05) and CRP (r = 0.3, p<0.05), IL-18 with smoking (0.4, p<0.001), MIC-1 with age (r = 0.4, p<0.001), smoking (r = 0.4, p<0.001), BMI (r = 0.4, p<0.01), SCORE (r = 0.3, p<0.05), Reynolds risk score (r = 0.4, p<0.05) and IMT (r = 0.3, p<0.05), TNF-R2 with ESR (r = 0.3, p<0.05), endoglin with ESR (r = -0.3, p<0.05) and DAS28 (r = -0.4, p<0.05), sICAM-1 with Reynolds risk score (r = 0.6, p<0.001) and IMT (r = 0.3, p<0.05) as well as sVCAM-1 with Reynolds risk score (r = 0.4, p<0.05).

**Fig 2:**
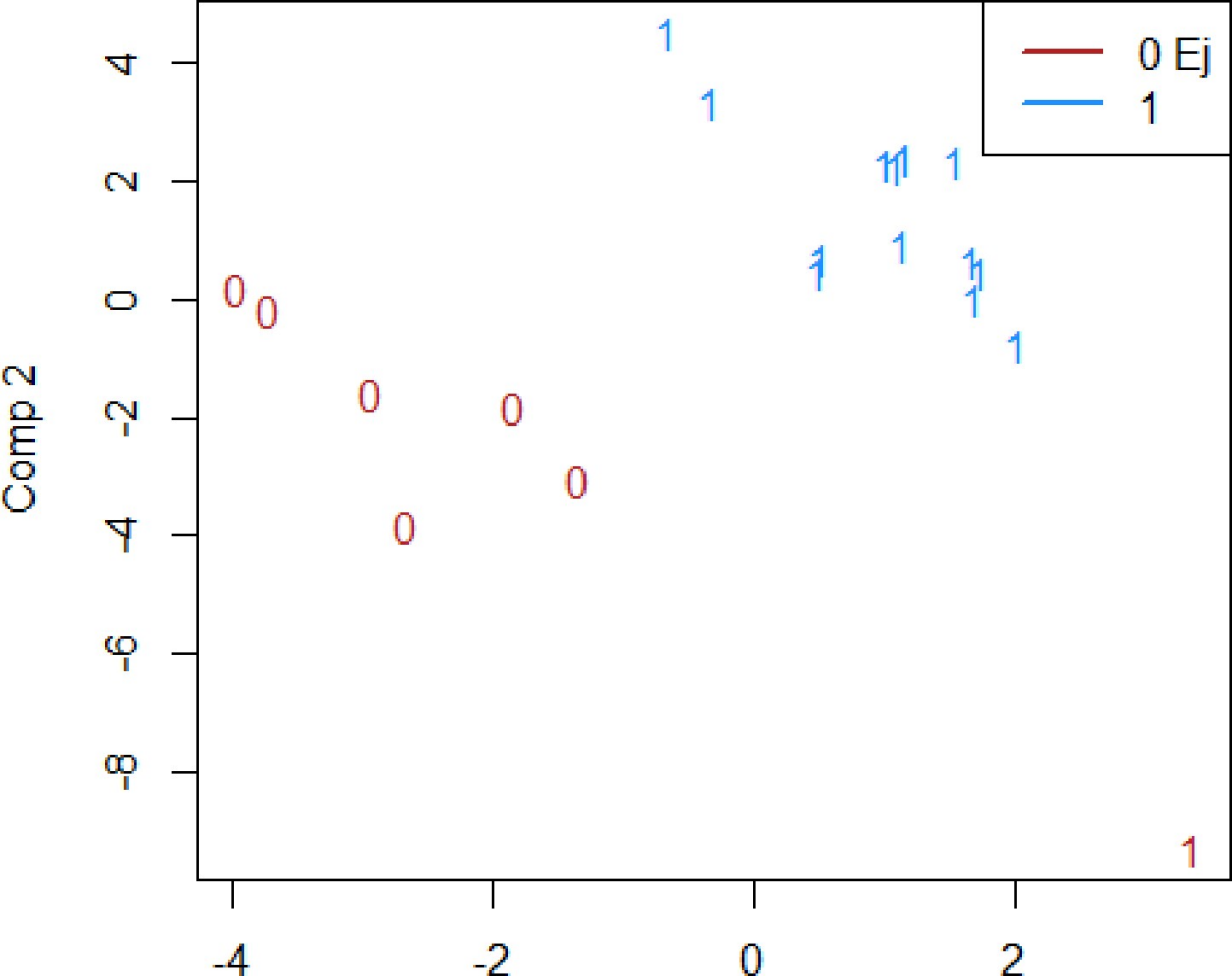
Classification by k-means as well as the true class for an observation for those who had a worsening in their IMT over the five years of follow up or not in a pilot study of 19 patients with RA. **The numbers indicate the true class and the colour the class according to the classification**. Portion of correctly classified with k-means clustering: 0.95. Portion of correctly classified with lda: 0.67. Variables of importance according to lasso: (Intercept), MMP3.

**Fig 3:**
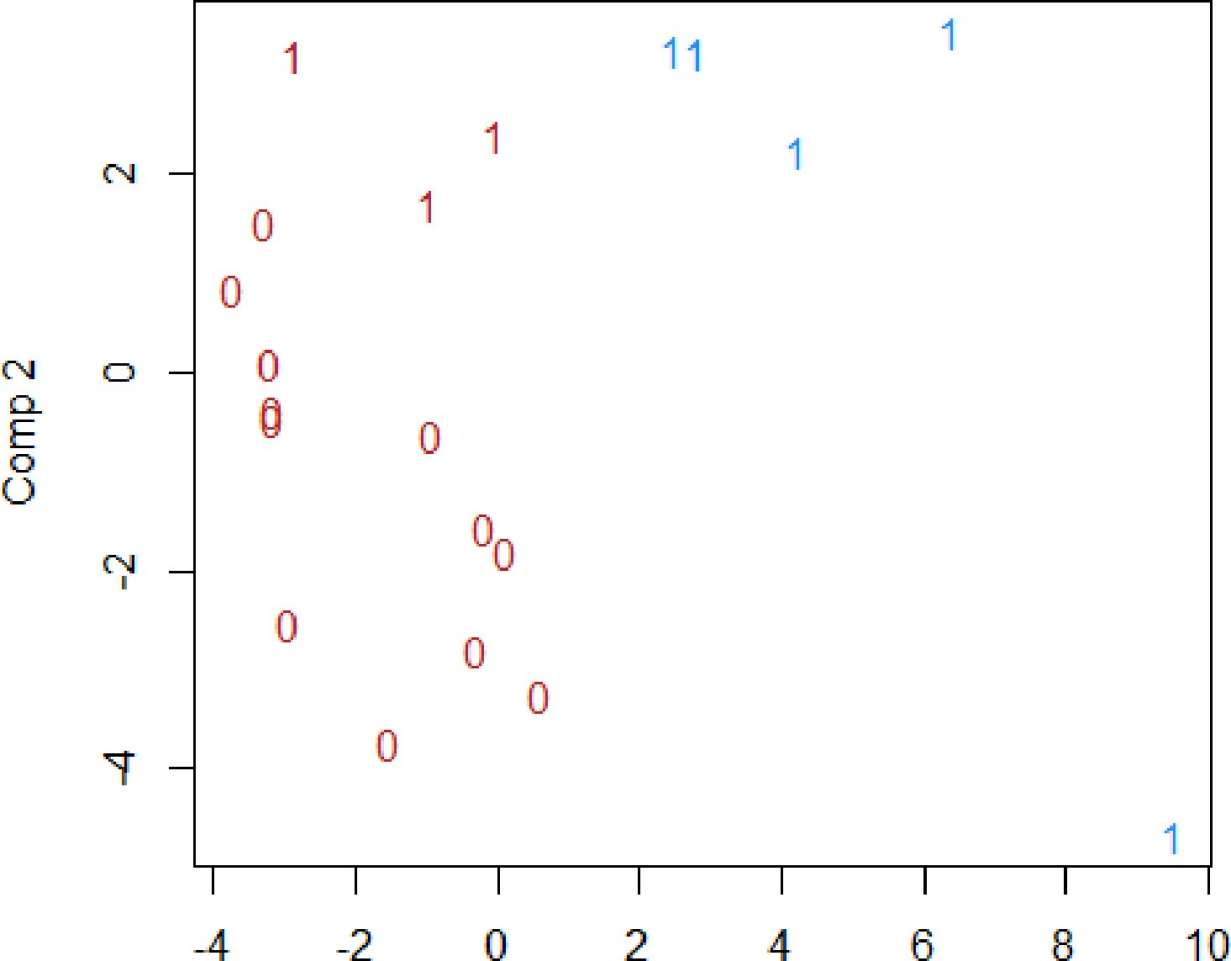
Classification by k-means as well as the true class for an observation for IMT over or under median value in a pilot study of 19 patients with RA. **The numbers indicate the true class and the colour the class according to the classification.** Portion of correctly classified with k-means clustering: 0.85. Portion of correctly classified with lda: 0.67. Variables of importance according to lasso: (Intercept).

Using PLSR on the CVD-panel analysed with multiplex, the patients with RA could be correctly classified into those who had a worsening in their IMT over the five years of follow up or not (Fig 2). Here, MMP3 was identified as influential. However, using data from the same CVD-panel, the patients with RA and IMT at T5 over/under median value could not be classified correctly with the biomarkers included (Fig 3). Also, none of the included biomarkers was identified as influential.

### Associations between biomarkers and IMT

In simple regression analysis, none of the biomarkers were associated with IMT at T0 but some at T5 (Table 3). Only MIF was significantly associated with the change in IMT between T0 and T5 (β = -0.01, 95%CI [-0.0001;-0.001], p-value = 0.036), although this association was not significant after adjustment for sex and age.

**Table 3:** Simple regression analysis with IMT at T5 as dependent variable and biomarkers important for CVD as covariates in 71 patients with early RA.

	β	95%CI	p-value
sCD40L at T0	0.1/ pg/mL	0.1;0.2	0.04^x^
sICAM-1 at T5	0.2/ ng/mL	0.00001;0.3	0.02^x^
MIC-1 at T5	0.04/ ng/mL	0.0001;0.1	0.01^x^

^x^ None of these associations remained statistically significant after adjustment for sex and age

## Discussion

Biomarkers of systemic inflammation, pro-inflammatory cytokines and chemokines have been suggested to be closely related to the atherosclerotic process. In this study we aimed to investigate some of these biomarkers, known to be associated with atherosclerosis and CVD in the general population, in a well characterised inception cohort of patients with early RA with data on prospective development of atherosclerosis. We found most of these potential biomarkers to be affected by the RA-disease itself, and therefore hard to identify as biomarkers of atherosclerosis in this setting. Among patients with RA, the area of surrogate endpoints is also difficult to interpret since several biomarkers of CVD are associated with general inflammation. Atherosclerosis is nowadays recognized as an inflammatory disease and there are many parallels between the pathological and immunological processes that occur in the synovium and the atheromatous lesions in the vessel walls [6]. A previous published study on the same individuals as in this study has suggested lipoprotein-associated phospholipase A2 (Lp-PLA2) as one of the biomarkers that might be able to indicate that, over time, the deleterious disease process in RA may also affect the vascular walls [16].

In this study, a few of the measured biomarkers that were associated with subclinical atherosclerosis. One of these, with significant different levels in patients with RA compared with controls, was MIF, that was significantly lower among patients with RA at T0, and also decreased over the five years of follow up, both in patients with RA and controls. Our results are contrary to most other published studies since MIF is a cytokine with pleiotropic roles in both acute and chronic inflammatory diseases suggested to be associated with the progression and severity of atherosclerotic disease [22, 23].

Metalloproteinases are all suggested to be markers of vulnerable plaques [14] and in this study the biomarker of influence in separating those with and without progression of the atherosclerosis in a subgroup of the patients with RA was a metalloproteinase, MMP3. However, MMP3 is also involved in cartilage degradation and bone destruction in RA [24, 25], again pointing out the interlink between the diseases in the joint and the vessel wall.

Some of the other biomarkers showed statistically significant differences between patients with RA and controls or were correlated with disease activity. Most well-known are the cellular adhesion molecules (CAMs) that mediate adhesion of circulating leukocytes to endothelial cells, promoting subsequent transendothelial migration and formation of atheroma. In this study both ICAM-1 and VCAM-1 were correlated with risk for future CVD and ICAM-1 also with subclinical atherosclerosis, in line with previous studies [14]. Also the pro-inflammatory cytokine IL-18 was significantly higher among patients with RA compared with controls at baseline, in line with previous studies where IL-18 is suggested as a mediator of inflammation and angiogenesis in the joints in RA [26]. In this study, IL-18 was also associated with some traditional CVD risk factors, again supporting a link between these two processes [26]. Another biomarker indicating the same link is MIC-1, previously suggested to be a marker of erosive joint disease in RA [27] and also suggested to be associated with a common etiologic of RA and CVD [28]. In this study the levels of MIC-1 was higher in patients with RA compared with controls and associated with several traditional CVD risk factors. Another soluble biomarker regarded as a marker of plaque instability is sCD40L, that is produced by activated platelets [14] and regarded as marker of CVD risk in the general population [29]. In previous studies the levels of sCD40L, have been shown to be elevated in patients with RA [30, 31]. The association to CVD in patients with RA is still a matter of discussion [28, 30, 31]. In this study the level of sCD40L was correlated with disease activity at baseline and also associated with IMT five years later. The complicated and possible complex relationship between these biomarkers and inflammation is further illustrated in this study where the difference between patients with RA and controls changed over time, most evident for IL-18, MIF and pentraxin 3.

The main strength of the present study is its prospective design from disease onset. Data were collected from the onset of disease and then continuously during five years of follow up. In northern Sweden, practically all of the patients with newly diagnosed RA are included in a structured follow up programme. Of these patients, all aged ≤60 years were invited to participate in the present study within 12 months of their diagnosis.

The main limitation is the number of controls, and unfortunately it was not possible to include more controls. Furthermore, no variables of inflammation were available among the controls. The associations between these biomarkers and CVD must, however, be regarded as well studied in the general population. This study was directed at the interplay between biomarkers and other CVD risk factors among the patients with RA.

In conclusion, this study indicates that the RA disease itself might affect several of the biomarkers in this study, and possible also the processes involved in the development of atherosclerosis. Subsequently, none of the biomarkers in this study could explain the burden of subclinical atherosclerosis in these patients with early RA. Nevertheless, further studies are important for a better understanding of the premature atherosclerotic process in patients with inflammatory diseases like RA.

## Supporting information

S1 Table Correlations between different biomarkers important for CVD in 71 patients with early RA and in 40 age and sex-matched controls evaluated at both baseline (T0) and after 5 years (T5). (DOCX)
