# Supplementary material for: Biomarkers associated with cardiovascular disease in patients with early rheumatoid arthritis
Source: PLoS One. 2019 Aug 5;14(8):e0220531. doi: 10.1371/journal.pone.0220531 (PMC6681963; doi:10.1371/journal.pone.0220531)
Supplement: S1 Table — (DOCX) [file pone.0220531.s001.docx]

|  | RA | |
| --- | --- | --- |
|  | T0 | T5 |
| IL-6 | sCD40L r=0.5*** | sCD40L r=0.4** |
| MPO | MIF r=0.3** | MIF r=0.5*** |
|  |  | MIC1 r=0.4*** |
|  |  | sCD40L r=0.4** |
| MIC1 |  | sCD40L r=0.3* |
|  |  | IL-18 r=0.5*** |
| pentraxin3 |  | VCAM-1 r=0.2** |
| TNF-R1 |  | endostatin r=0.4** |
|  |  | VCAM-1 r=0.4*** |
|  |  | ICAM-1 r=0.4*** |
| endoglin |  | VCAM-1 r=0.3* |
| ICAM-1 |  | endostatin r=0.4** |
|  |  | VCAM-1 r=0.4*** |
